# Evaluating the Oral Hygiene and Periodontal Status of Patients Undergoing Hemodialysis

**DOI:** 10.7759/cureus.80086

**Published:** 2025-03-05

**Authors:** Shilpa Ramachandran, Rajesh Vyloppillil, Rajesh Nair, Pallavi Menon

**Affiliations:** 1 Department of Periodontics, Amrita School of Dentistry, Amrita Vishwa Vidyapeetham, Kochi, IND; 2 Department of Nephrology, Amrita Institute of Medical Sciences, Amrita Vishwa Vidyapeetham, Kochi, IND

**Keywords:** hemodialysis, oral hygiene, oral prophylaxis, periodontal disease, periodontal parameters

## Abstract

Background

Periodontal disease is a chronic inflammatory condition affecting the periodontium, which is induced by various pathogenic microorganisms. The severity and frequency of periodontal disease among patients undergoing hemodialysis are higher compared to healthy individuals. This study aimed to evaluate whether oral prophylaxis and proper oral hygiene maintenance help improve the periodontal condition of patients undergoing hemodialysis.

Methodology

In this study, 30 participants satisfying the inclusion criteria were selected. We assessed the oral hygiene (Oral Hygiene Index Simplified (OHI-S)) and the periodontal status (probing pocket depth (PPD) and clinical attachment level (CAL)) of the included participants. Non-surgical periodontal management comprising oral prophylaxis and oral hygiene maintenance instructions were provided one week before initiating hemodialysis. The patients were then re-examined after one and three months. At each visit, oral hygiene status and clinical periodontal examinations were performed, along with reinforcing oral hygiene instructions to all participants.

Results

The mean age of the study participants was 44.10 ± 5.02 years. Repeated-measures one-way analysis of variance was used to compare the mean OHI-S, CAL, and PPD at different periods (baseline, after one month, and after three months), followed by multiple comparison using Bonferroni test. There was a statistically significant improvement in oral hygiene status and periodontal parameters (PPD and CAL) after one month and three months (p < 0.001) while the patient continued with hemodialysis.

Conclusions

This study showed the potential benefits of oral prophylaxis, oral hygiene maintenance, and periodontal care in patients undergoing hemodialysis.

## Introduction

Periodontal disease is a group of inflammatory pathologies affecting the supporting structures of the teeth [1]. Chronic renal failure is a progressive disease characterized by the destruction of nephrons and subsequent reduction in kidney function [2]. Earlier, periodontal disease and end-stage renal disease (ESRD) were thought to be not associated because of their difference in pathogenesis, as periodontitis is a localized infection caused by microbial biofilm and ESRD is a condition where the kidneys function below 10% of their normal function [3,4]. However, several studies have shown the close and complex link between periodontal disease and chronic kidney disease. Some studies suggest that ESRD has adverse effects on periodontal health [5-9], whereas some support the adverse effects of periodontitis on ESRD [7,10]. Hemodialysis is a form of renal replacement therapy, wherein the toxic waste and excessive proteins and electrolytes, such as urea and potassium, are removed from the blood. Reports suggest that 99.1% of hemodialysis patients exhibit some form of periodontal disease [4,11,12]. Several studies have also reported that over half of hemodialysis patients exhibit periodontal disease [13,14]. Non-surgical periodontal management is a simple therapeutic approach which can reduce periodontal infections, thereby improving the systemic status [15-18].

Thus, in this study, we hypothesized that non-surgical periodontal management can have beneficial effects on the systemic condition of patients undergoing hemodialysis. However, due to lack of evidence, it remains unclear to what extent non-surgical periodontal management would impact ESRD patients. Hence, our study aimed to evaluate whether oral prophylaxis and proper oral hygiene maintenance help improve the periodontal status of patients undergoing hemodialysis.

Objectives of the study

The primary objective was to assess and compare the oral hygiene status of patients in the age group of 30 to 60 years before hemodialysis and after one and three months. The secondary objective was to assess and compare the periodontal status of patients in the age group of 30 to 60 years before hemodialysis and after one and three months.

## Materials and methods

This study was conducted in the Department of Nephrology, Amrita Institute of Medical Sciences and the Department of Periodontics, Amrita School of Dentistry, Kochi, India from June 6, 2018, to June 15, 2019. The study was approved by the Ethical Committee of Amrita School of Dentistry, Amrita Vishwa Vidyapeetham, Kochi, Kerala (approval number: IRB-AIMS-2018-076).

The study participants were patients reporting to the Department of Nephrology, Amrita Institute of Medical Sciences diagnosed with chronic kidney disease and requiring hemodialysis. The inclusion criteria were patients indicated for hemodialysis in the age group of 30 to 60 years, those having at least 16 natural teeth, and non-smokers who were willing to participate in the study. The exclusion criteria included patients with any malignancy, those with diabetes mellitus, those on antibiotics and immunosuppressants, and those on any anti-inflammatory drugs.

Study design

In this short interventional study, all participants meeting the selection criteria were numbered from 1 to 30 according to the recruitment sequence. Individuals participating in the study were explained about the nature of the study, following which verbal and written informed consent was obtained. Non-surgical periodontal management consisting of oral hygiene instructions and supragingival and subgingival scaling was performed in these patients at the department of periodontics by a single periodontist using an ultrasonic scaler with fine tips. The procedure was completed without administration of antibiotics or local anti-microbials at baseline. Assessment of oral hygiene and periodontal status of the same patients one week before the initiation of hemodialysis was performed. The patients were then re-examined after one and three months. At each visit, a detailed clinical periodontal examination was performed and the oral hygiene status was determined with the reinforcement of the oral hygiene instructions to all participants by the same periodontist. The examiner could not be blinded to the subject’s general condition as they were examined in a hospital. The clinical oral examination was performed under artificial light with the use of a mouth mirror and a Williams graduated periodontal probe. The oral hygiene condition was assessed using the Oral Hygiene Index Simplified (OHI-S) [19] on six indexed teeth, and the periodontal status was assessed using the clinical attachment loss (CAL) as per the World Health Organization methodology.

Statistical analysis

All statistical analysis was performed using SPSS version 20 software (IBM Corp., Armonk, NY, USA). Data were presented as mean ± standard deviation (SD), and 95% confidence interval (CI) for continuous parameters and as frequency and percentage for categorical data. Based on the mean and SD of OHI-S at less than one year (3.23 ± 1.38), observed in an earlier study [18] and with 95% CI and 15% allowable error (alpha error and relative precision was considered), the study was conducted with a minimum sample size of 30 participants. Pearson’s correlation coefficient was used to determine the correlation between age and OHI-S and periodontal parameters (CAL and probing pocket depth (PPD)). Independent-sample t-test was applied to determine the statistically significant difference in the mean OHI-S, CAL, and PPD between male and female participants. Repeated-measures one-way analysis of variance was used to compare the mean OHI-S, CAL, and PPD at different periods (baseline, after one month, and after three months), followed by multiple comparison using Bonferroni test. In this study, there are three time points, and as we performed three pairwise comparisons, the Bonferroni correction was calculated as 0.05/3 = 0.0167. A p-value <0.05 was considered a statistically significant difference.

## Results

A total of 30 participants were included in the study, with 17 males and 13 females. The age range of the participants was 30-60 years, with the mean age being 44.10 ± 5.02 years (Table 1).

**Table 1:** Characteristics of study participants.

Variable	Number
Total number of participants	30
Male	17
Female	13
Mean age	44.10 ± 5.02 years

Table 2 shows the comparison of OHI-S at baseline, after one month, and after three months. There was a statistically significant difference when the mean OHI-S at the different periods (baseline, after one month, and after three months) were compared (p < 0.001).

**Table 2:** Comparison of OHI-S before the initiation of hemodialysis (baseline) and after one and three months. P-value <0.05 was considered statistically significant; repeated-measures one-way analysis of variance. OHI-S = Oral Hygiene Index Simplified; SD = standard deviation

Visit	OHI-S (mean ± SD)	P-value
Baseline	3.42 ± 1.19	<0.001
1 month	2.82 ± 1.08	
3 months	2.19 ± 1.01	

Table 3 shows the comparison of CAL before the initiation of hemodialysis and after one month and three months. A statistically significant difference in mean CAL was noted (p < 0.001).

**Table 3:** Comparison of CAL before the initiation of hemodialysis (baseline) and after one and three months. P-value <0.05 was considered statistically significant; repeated-measures one-way analysis of variance. CAL = clinical attachment level; SD = standard deviation

Visit	CAL (mean ± SD)	P-value
Baseline	7.16 ± 1.08	<0.001
1 month	6.66 ± 1.15	
3 months	6.06 ± 1.28	

Table 4 compares the PPD at different time intervals. A statistically significant relation was noted with a p-value <0.001.

**Table 4:** Comparison of PPD before the initiation of hemodialysis (baseline) and after one and three months. P-value <0.05 was considered statistically significant; repeated-measures one-way analysis of variance. PPD = probing pocket depth; SD = standard deviation

Visit	PPD (mean ± SD)	P-value
Baseline	6.60 ± 1.276	<0.001
1 month	6.23 ± 1.406	
3 months	5.80 ± 1.562	

Table 5 shows the correlation between age and OHI-S. There was a negative correlation between age and OHI-S.

**Table 5:** Correlation between age and OHI-S. r = correlation coefficient; Pearson’s correlation coefficient. P-value <0.05 was considered statistically significant. OHI-S = Oral Hygiene Index Simplified

OHI-S	r value	P-value
Baseline	-0.095	0.619
1 month	-0.138	0.468
3 months	-0.147	0.438

Table 6 shows the correlation between age and CAL. There was no significant correlation between age and CAL.

**Table 6:** Correlation between age and CAL. r = correlation coefficient; Pearson’s correlation coefficient. P-value <0.05 was considered statistically significant. CAL = clinical attachment level

CAL	r value	P-value
Baseline	-0.214	0.256
1 month	-0.250	0.183
3 months	-0.195	0.301

Table 7 shows the correlation between age and PPD. The r-value showed no significant correlation between age and PPD.

**Table 7:** Correlation between age and PPD. r = correlation coefficient; Pearson’s correlation coefficient. P-value <0.05 was considered statistically significant. PPD = probing pocket depth

PPD	r value	P-value
Baseline	0.092	0.627
1 month	0.138	0.467
3 months	0.047	0.807

Table 8 shows the comparison between gender and OHI-S. The p-value of OHI-S at baseline, after one month, and after three months concerning the gender were 0.938, 0.932, and 0.707, respectively. There was no statistically significant correlation between gender and OHI-S.

**Table 8:** Comparison between gender and OHI-S. Significance (two-tailed): independent-sample t-test. OHI-S = Oral Hygiene Index Simplified; SD = standard deviation; M = male; F = female

OHI-S	Gender	Mean ± SD	Sig. (two-tailed)
Baseline	M	3.43 ± 1.17	0.938
	F	3.40 ± 1.27	
1 month	M	2.83 ± 1.13	0.932
	F	2.80 ± 1.07	
3 months	M	2.25 ± 0.99	0.707
	F	2.11 ± 1.06	

Table 9 shows the comparison between gender and CAL. The results showed no statistically significant relationship between gender and CAL.

**Table 9:** Comparison between gender and CAL. Significance (two-tailed): independent-sample t-test. CAL = clinical attachment level; SD = standard deviation; M = male; F = female

CAL	Gender	Mean ± SD	Sig. (two-tailed)
Baseline	M	9.12 ± 1.79	0.958
	F	9.08 ± 2.39	
1 month	M	8.71 ± 1.92	0.793
	F	8.92 ± 2.56	
3 months	M	8.35 ± 2.06	0.905
	F	8.46 ± 2.90	

Table 10 shows the comparison between gender and PPD. The results showed no statistically significant relationship between gender and PPD.

**Table 10:** Comparison between gender and PPD. Significance (two-tailed): independent-sample t-test. PPD = probing pocket depth; SD = standard deviation; M = male; F = female

PPD	Gender	Mean ± SD	Sig. (two-tailed)
Baseline	M	6.71 ± 1.21	0.612
	F	6.46 ± 1.39	
1 month	M	6.24 ± 1.25	0.993
	F	6.23 ± 1.64	
3 months	M	5.94 ± 1.29	0.580
	F	5.62 ± 1.89	

Table 11 shows the pairwise comparison at baseline, after one month, and after three months.

**Table 11:** Pairwise comparison at baseline and after one month and three months. Post-hoc Bonferroni test. Bonferroni correction ∝ = 0.0167. * = the mean difference is significant at the 0.05 level. ^b^ = adjustment for multiple comparisons: least significant difference (equivalent to no adjustments).

Pairwise comparisons		Mean difference (I-J)	Standard error	Sig.^b^	95% confidence interval for difference^b^	
					Lower bound	Upper bound
Baseline	1 month	0.500^*^	0.104	0.000	0.286	0.714
	3 months	1.100^*^	0.130	0.000	0.834	1.366
1 month	Baseline	-0.500^*^	0.104	0.000	-0.714	-0.286
	3 months	0.600^*^	0.091	0.000	0.414	0.786
3 months	Baseline	-1.100^*^	0.130	0.000	-1.366	-0.834
	1 month	-0.600^*^	0.091	0.000	-0.786	-0.414

## Discussion

Periodontal disease is a chronic inflammatory condition that leads to the formation of periodontal pockets, destruction of the deeper collagenous structures of the periodontium and alveolar bone, excessive tooth mobility, and, eventually, early tooth loss [20]. This infectious disease is caused by Gram-negative bacteria that induce a systemic inflammatory response [21]. The incidence of ESRD continues to rise worldwide, and, in India, it is estimated that 1,650,000‑2,200,000 people out of 1.1 billion develop ESRD annually, increasing the number of individuals with ESRD who will require oral health care [22].

Non-surgical periodontal therapy is the cornerstone of periodontal disease management, and it is the first recommended approach to control periodontal infection [23]. Some studies applied more radical therapies to reduce periodontal inflammation, such as pocket-reduction therapy and local drug delivery [24,25]. The present study did not include these treatment modalities because we intended to assess the effectiveness of non-surgical periodontal therapy alone.

The oral hygiene and periodontal status of 30 patients undergoing hemodialysis were analyzed in this study. Oral prophylaxis was performed before the initiation of hemodialysis, and the brushing technique (modified Bass technique) was demonstrated to patients who were asked to follow it. Patients were recalled after one week before the first hemodialysis, and the oral hygiene status was recorded by determining OHI-S. Periodontal status was recorded by measuring CAL and PPD. The patients were then recalled after one month and three months, and oral hygiene and periodontal status were recorded again.

The present study was done in accordance with the studies conducted by Davidovich et al. [26], Naugle et al. [5], Bayraktar et al. [8], Murthy and Hiremath [27], and Sobrado Marinho et al. [28], which showed that the oral hygiene and periodontal status worsen with the duration of hemodialysis with the highest community periodontal index and loss of attachment score. This might be because patients receiving dialysis are preoccupied with their main life-threatening problem and they neglect basic oral care.

In this study, oral prophylaxis followed by oral hygiene instructions and brushing technique were demonstrated to the study subjects before the initiation of hemodialysis, and their oral hygiene and periodontal status were recorded as baseline parameters after one week. Subsequently, the same parameters were recorded after one month and three months while the patient continued with hemodialysis. At each dental visit, oral hygiene maintenance was reinforced. The results of this study showed a statistically significant improvement in OHI-S and clinical parameters such as CAL and PPD (p < 0.001).

Age is the known risk factor for both chronic kidney diseases and periodontal diseases [29]. The mean age of the dialysis patients in this study was 44.10 ± 5.026, which was higher than the subjects of a previous study [30].

In the present study, the periodontal parameters CAL and PPD reduced during the follow-up period. Hence, patients should be advised about proper oral hygiene maintenance and should be frequently monitored by the dentist. No significant correlation was found between age, gender, and the oral hygiene status and the periodontal parameters examined. The findings support findings of Naugle et al., suggesting that individuals on dialysis do not receive adequate periodontal care and the disease progresses unchecked [5]. There is a paucity of literature regarding the effect of oral prophylaxis on oral hygiene and periodontal condition in hemodialysis patients. To our knowledge, this study is one of the pioneer studies evaluating the periodontal status in hemodialysis patients after providing non-surgical periodontal therapy.

Clinical implications

Patients undergoing hemodialysis are at a higher risk for oral and periodontal diseases due to several factors, including xerostomia, compromised immune function, and the potential for systemic infections. Periodontal disease in these patients may lead to further complications, including cardiovascular diseases and other infections. Chronic periodontal inflammation can elevate systemic inflammatory markers, which may have a negative impact on the patient’s kidney function. Thus, regular evaluation of oral hygiene and periodontal status is crucial in maintaining overall health. The results of our study showed that there was a reduction in the periodontal parameters, such as PPD and CAL, as well as an improvement in the oral hygiene status with proper follow up. The study also showed the significance of an interdisciplinary approach between periodontists and nephrologists, demonstrating how collaborative care plays a significant role in managing the patient’s overall health and reducing the impact of periodontal diseases on renal function.

Study limitations

The study findings should be further validated by conducting long-term follow-up studies on larger samples, which may provide more robust data. However, recruiting enough hemodialysis patients who are willing to attend recall visits presents a significant challenge.

## Conclusions

Several studies have shown that longer duration of hemodialysis is associated with severe periodontal diseases. The present investigation showed that periodontal therapy reduced the inflammatory component and was indicated as an important interventional therapy for patients undergoing hemodialysis. Within the limitation of our study, we concluded that oral prophylaxis and early dental care, which includes good oral hygiene practices comprising proper tooth brushing, flossing, and regular dental visits preferably every three months for reinforcement of oral hygiene practices to control gingival inflammation, should be intensified in chronic renal failure patients, which can, in turn, play a positive role in improving their general health status.
